# ACS network-based implementation of therapeutic hypothermia for the treatment of comatose out-of-hospital cardiac arrest survivors improves clinical outcomes: the first European experience

**DOI:** 10.1186/1757-7241-21-22

**Published:** 2013-03-25

**Authors:** Marek Kozinski, Krzysztof Pstragowski, Julia Maria Kubica, Tomasz Fabiszak, Michal Kasprzak, Blazej Kuffel, Przemyslaw Paciorek, Eliano Pio Navarese, Grzegorz Grzesk, Jacek Kubica

**Affiliations:** 1 Department of Cardiology and Internal Medicine, Collegium Medicum, Nicolaus Copernicus University, Bydgoszcz, Poland; 2 Students’ Scientific Society, Department of Cardiology and Internal Medicine, Collegium Medicum, Nicolaus Copernicus University, Bydgoszcz, Poland; 3 Department of Pharmacology and Therapeutics, Collegium Medicum, Nicolaus Copernicus University, Bydgoszcz, Poland; 4 Depertment of Emergency Medicine, Collegium Medicum, Nicolaus Copernicus University, Bydgoszcz, Poland

**Keywords:** Hypothermia, Cardiac arrest, Regional system of care, Stent thrombosis

## Abstract

**Background:**

There is a paucity of data regarding clinical outcomes associated with the integration of a mild therapeutic hypothermia (MTH) protocol into a regional network dedicated to treatment of patients with acute coronary syndromes (ACS). Additionally, a recent report suggests that the neurological benefits of MTH therapy in interventionally managed ACS patients resuscitated from out-of-hospital cardiac arrest (OHCA) may be potentially offset by the catastrophic occurrence of stent thrombosis. The goal of this study was to share our experience with the implementation of an MTH program using a previously established ACS network in consecutive comatose OHCA survivors undergoing interventional management due to an initial diagnosis of ACS and to assess the clinical effectiveness and safety of MTH.

**Methods:**

We conducted a retrospective historically controlled single centre study. Hospital survival with a favourable neurological outcome (Cerebral Performance Category of 1 or 2) and all-cause in-hospital mortality were the primary and secondary efficacy end points, respectively. Occurrence of definite stent thrombosis was the primary safety end point while the development of pneumonia, presence of positive blood cultures, occurrence of probable stent thrombosis, any bleeding complications, need for red blood cell transfusion and presence of rhythm and conductions disorders during hospitalisation constituted secondary safety end points.

**Results:**

Comatose OHCA survivors (n = 32) were referred to our Department based on ECG recording transmissions and/or phone consultations or admitted from the Emergency Department. Compared with controls (n = 33), they were significantly more likely to be discharged from hospital with a favourable neurological outcome (59 vs. 27%; p < 0.05; number needed to treat [NNT] = 3.11) and experienced lower all-cause in-hospital mortality (13 vs. 55%; p < 0.05; NNT = 2.38). Rates of all safety end points were similar in patients treated with and without MTH.

**Conclusions:**

Our study indicates that a regional system of care for OHCA survivors may be successfully implemented based on an ACS network, leading to an improvement in neurological status and to a reduction of in-hospital mortality in patients treated with MTH, without any excess of complications. However, our findings should be verified in large, prospective trials.

## Introduction

Cardiac arrest constitutes the most frequent reason for sudden death in developed countries. It is estimated that each year 275,000 and 235,000 to 325,000 new cases of out-of-hospital cardiac arrest (OHCA) occur in Europe and the United States, respectively [1,2]. The rate of admission to hospital after a successful return of spontaneous circulation in adult OHCA patients is estimated to be around 15% [3]. Although the survival of adult OHCA victims admitted to hospital has increased recently, approximately 60% of these patients still die before hospital discharge [4]. Furthermore, only two thirds of OHCA survivors are discharged from hospital in a good neurological condition [5].

Mild therapeutic hypothermia (MTH) remains one of few interventions proven to improve clinical outcomes in the setting of OHCA [6,7]. Based on evidence from randomized clinical trials [6-9], the Advanced Life Support Task Force of the International Liaison Committee on Resuscitation in 2003 [10] and subsequently The European Resuscitation Council in 2005 [11] recommended MTH in unconscious adult patients with recovery of spontaneous circulation after OHCA when the initial rhythm was ventricular fibrillation. Additionally, according to these recommendations MTH may also be beneficial for other rhythms or in-hospital cardiac arrest [10,11]. Finally, the role of MTH was appreciated by the European Society of Cardiology in 2012 [12]. The guidelines for the management of acute myocardial infarction in patients presenting with ST-segment elevation indicate implementation of MTH “early after resuscitation of cardiac arrest patients who are comatose or in deep sedation” (class of recommendation I, level of evidence B).

On the contrary, some international experts express the opinion that MTH due to the substantial risk of bias in randomized clinical trials and their overall small size and low quality should be further evaluated [13,14]. Additionally, even though a recent meta-analysis of randomized clinical trials conducted by the Cochrane Collaboration demonstrated impressive efficacy of MTH (the number needed to treat [NNT] of 6 for discharge in a good neurological condition; NNT of 7 for survival to hospital discharge) [15], this therapeutic method is still underutilized in many European countries including Poland [16,17]. This fact stands in apparent contrast to the high penetration of interventional treatment in the setting of acute coronary syndromes (ACS) and the presence of well-developed ACS networks in these countries [18]. Of major importance, vast majority of patients from landmark randomized clinical trials on MTH were treated conservatively, despite the dominating presumed cardiac origin of OHCA [6,7]. There is a paucity of data regarding clinical outcomes associated with the integration of MTH protocols into regional networks dedicated to treatment of ACS patients. A recent observational American report suggests that such management leads to a favourable prognosis in a substantial proportion of comatose OHCA patients [19]. On the other hand, the neurological benefits of MTH therapy in interventionally managed ACS patients may be potentially offset by the catastrophic occurrence of stent thrombosis as suggested by the just published case series from Barcelona [20].

The goal of this study is to share our experience regarding the implementation of an MTH program using a previously established ACS network in comatose OHCA survivors undergoing interventional management due to an initial diagnosis of ACS and to assess the clinical effectiveness and safety of MTH.

## Methods

### Study design

We conducted a retrospective historically controlled single centre study. Consecutive adult comatose OHCA survivors with an initial diagnosis of ACS who received MTH therapy between 2010 and 2011 were compared with their consecutive counterparts fulfilling all criteria for therapy with MTH, but not treated with this method, as they were hospitalized before implementation of the MTH program, namely in 2008 and 2009.

Qualification criteria for MTH included: unconsciousness with no verbal contact, Glasgow Coma Score below 9 points, age of at least 18 years, and eligibility for mechanical ventilation. Exclusion criteria for MTH were as follows: Glasgow Coma Score equal or above 9 points, maintained verbal contact, vegetative state or severe brain damage before OHCA, history of severe infection prior to OHCA, all prior conditions with estimated length of survival below one year, active bleeding unresponsive to treatment, active intracranial bleeding, bleeding diathesis unless induced with drugs, pregnancy (ruled out in all women below 50 years of age with a pregnancy test; however, after the update of the European Resuscitation Council guidelines in December 2010 [21] early gestation was no longer regarded as a contraindication for MTH), hemodynamic instability defined as systolic arterial pressure below 65 mm Hg despite therapy with vasopressors and/or application of intraaortic balloon counterpulsation (IABP) with reassessment after hemodynamic stabilization, electrical instability defined as the need for defibrillation or cardioversion within 30 minutes preceeding qualification for MTH (with reassessment once patient electrically stable), and symptomatic bradyarrhythmias unresponsive to endocardial pacing.

### Study conduction and MTH procedure

OHCA survivors were referred to our Department similarly to other ACS patients either based on a telephone consultation predominantly accompanied by an ECG transmission through the LIFENET System (Medtronic, Redmond, WA, USA) from the site of OHCA occurrence or admitted directly from the Emergency Department of our hospital.

Adult patients were qualified to therapy with MTH regardless of the initial rhythm during OHCA, time to return of spontaneous circulation and age. The baseline neurologic assessment was routinely performed twice within the initial 30 minutes of the qualification procedure, and optionally followed whenever feasible by further neurologic evaluation only when necessitated by prior administration of sedatives and their antidotes.

Patients with an initial diagnosis of ACS with ST-segment elevation and/or in shock of clear cardiac origin were transferred directly from the ambulance, rescue helicopter or Emergency Department to the cathlab to avoid any delay in revascularization. The decision concerning qualification to therapy with MTH was made immediately after the patient’s admission. Subsequently, induction of MTH was initiated with the infusion of cold normal saline (0.9% solution of sodium chloride at the temperature of 4°C) and the usage of ice packs.

All patients from both the study and control group underwent coronary angiography, followed, if clinically indicated, by percutaneous coronary intervention (PCI). Coronarography and PCI were performed using a standard femoral approach. Intracoronary stents were routinely implanted. Complete revascularization was intended in all patients with cardiogenic shock. Optimal direct effect of the intervention was assigned when no residual stenosis or a stenosis of less than 20% of the reference segment diameter along with TIMI (Thrombolysis in Myocardial Infarction) 3 flow in the PCI-treated artery were observed. Suboptimal direct effect of PCI was defined as the presence of a residual stenosis between 20 and 50% of the reference segment diameter and/or TIMI 2 flow the PCI-treated artery. A residual stenosis exceeding 50% of the reference segment diameter and/or TIMI 0 or 1 flow were considered ineffective effects of PCI. The use of aspiration thrombectomy during the intervention was left to the operator’s discretion.

Transthoracic echocardiography was performed in all study participants on the day of admission and thereafter whenever needed and serial ECG recordings were taken at baseline, after coronary angioplasty and/or coronary angiography and thereafter when clinically indicated. In case of any doubts regarding the reason of OHCA, additional diagnostic procedures including head and/or chest computed tomography were performed before the initiation of the MTH procedure.

An MTH-dedicated catheter was placed by the interventional cardiologist in the cathlab in the inferior vena cava through the femoral vein. The cooling system applied in our study was CoolGard 3000 (Alsius, Zoll, Chelmsdorf, MA, USA) allowing for precise and rapid control of the patient’s core temperature for both cooling and warming. The cooling procedure with the CoolGard 3000 system was started immediately after the patients had been transferred from the cathlab to Cardiac Intensive Care Sub-unit. The state of MTH was defined as the patient’s core temperature below 34°C, with a target temperature of 33°C. MTH was maintained for at least 12, with the optimal duration of MTH of 24 hours. The rewarming phase was actively controlled and performed at a rate of 0.3°C per hour. Two independent measurements of the patient’s core temperature were continuously taken in the urinary bladder and in the lower one third of the oesophagus using a dedicated catheter and tube, respectively.

All study participants were hospitalized in a 5-bed Cardiac Intensive Care Sub-unit that remains an integral part of the 24-bed Coronary Care Unit in our department. Medical care was delivered by cardiologists experienced in acute cardiac care and supported when needed by a multidisciplinary team of specialists available in an academic hospital, including anaesthesiologists, clinical microbiologists, clinical nutritionists, endoscopic gastroenterologists, general and vascular surgeons, medical rehabilitation specialists, nephrologists, neurologists and otolaryngologists.

All patients were mechanically ventilated and given continuous intravenous infusion of propofol and fentanyl for sedation and analgesia, respectively. Standard management of shivers included a combination of superficial rewarming, buspirone and magnesium, and was supplemented by atracurium when the shivers were resistant to the first-line therapy. All drugs were administered in a weight-adjusted manner.

All patients underwent continuous monitoring of invasive blood pressure, central venous pressure, ECG and hourly diuresis as well as multiple laboratory blood tests were performed according to our local MTH protocol (details available on request).

We aimed to achieve and maintain the hemodynamic and laboratory targets recommended by the International Liaison Committee for patients with post-cardiac arrest syndrome [22] such as mean arterial pressure of 65–100 mmHg (depending on the patient’s normal blood pressure, the cause of OHCA, and the severity of myocardial dysfunction), central venous pressure of 8–12 mmHg, central venous oxygen saturation above 70%, urine output >1 mL × kg^-1^ × h^-1^ and a normal or decreasing serum or blood lactate level.

The study protocol was approved by the Local Bioethics Committee at Collegium Medicum of the Nicolaus Copernicus University in Torun in accordance with the Declaration of Helsinki.

### Concomitant therapy

At the first contact with health care providers all study participants with a confirmed diagnosis of ACS were pretreated with an intravenous bolus of unfractionated heparin (70 IU/kg, up to 5000 IU) and oral loading doses of clopidogrel (600 mg) and aspirin (300 mg) administered as crushed tablets via nasogastric tube. In patients with an uncertain initial diagnosis loading with unfractionated heparin and antiplatelet agents was deferred until ACS confirmation. At the catheterization laboratory, patients undergoing PCI received second dose of unfractionated heparin intraarterially in a weight-adjusted manner (up to 100 IU/kg) or under activated clotting time guidance (to the target range of 200–250 seconds) when abciximab was intended. Therapy with unfractionated heparin under the activated partial thromboplastin time monitoring (with a therapeutic range of 1.5 to 2.5 times the control value) was continued in all study participants until the end of the rewarming phase. Then the patients were treated with prophylactic doses of enoxaparin until their full mobilization. Throughout the whole hospitalization aspirin was given to all patients with coronary artery disease at a dose of 75 mg q.d. Continuation of clopidogrel therapy was restricted to patients with a confirmed ACS diagnosis. Clopidogrel was administered at a dose of 150 mg q.d. until the end of the rewarming phase in MTH-treated patients and thereafter the dose was reduced to 75 mg q.d. while the controls received clopidogrel maintenance doses of 75 mg q.d. since the 2nd day until hospital discharge. Dual antiplatelet therapy was recommended for 12 months, unless the patient was considered to have a high risk of bleeding, with the minimal duration of 1 and 12 months after bare metal stent and drug-eluting stent implantation, respectively. Abciximab was given at the discretion of the invasive cardiologist. The drug was injected as an intracoronary bolus of 0.25 mg/kg with a subsequent intravenous infusion of 0.125 mg/min over 12 h according to the manufacturer’s recommendations.

If clinically indicated, norepinephrine was used as the vasopressor-of-choice to restore blood pressure and maintain adequate tissue perfusion. All study participants with a confirmed diagnosis of ACS received statin. Angiotensin-converting-enzyme inhibitors and beta-blockers were initiated in all patients on condition of sufficient values of blood pressure. Loop diuretics were started in case of congestion while aldosterone antagonists were given in patients with left ventricular ejection fraction below 35% and concomitant symptoms of heart failure or diabetes. All study participants received pantoprazole to prevent gastrointestinal bleedings. The combination of amoxicillin and clavulanic acid in doses adjusted to renal function was initiated immediately after admission in all subjects from the MTH group but not in patients from the control group. Therapy with antibiotics was modified or started (in the control group) in case of infection diagnosed based on the results of bronchial alveolar lavage, blood and urine cultures, inflammatory markers and/or clinical judgement.

All study participants received enteral nutrition in order to provide energy and nutrient intake as well as to preserve the gastrointestinal architecture and prevent bacterial translocation from the colon.

### Study endpoints

Hospital survival with a favourable neurological outcome and all-cause in-hospital mortality constituted the primary and the secondary efficacy end point, respectively. To evaluate the neurological status of the study participants at hospital discharge, we applied the Pittsburgh Cerebral Performance Category Scale [23] that remains a widespread and validated tool for the neurological assessment of patients treated with MTH [6,7,16]. It contains 5 categories: CPC-1 (good recovery), CPC-2 (moderate disability), CPC-3 (severe disability), CPC-4 (vegetative status) and CPC-5 (death). Survival with a favourable neurological outcome was defined as a Pittsburgh Cerebral Performance Category of 1 or 2, implicating that patients falling in these categories have sufficiently preserved brain function to live independently [6,7,23].

Occurrence of definite stent thrombosis was the primary safety end point while the development of pneumonia, presence of positive blood cultures, occurrence of probable stent thrombosis, any bleeding complications, need for red blood cell transfusion and presence of rhythm and conductions disorders during hospitalisation constituted the secondary safety end points. Definite stent thrombosis was defined as thrombosis within the stent confirmed angiographically or at autopsy. In the search of cases with probable stent thrombosis in patients with unexplained in-hospital death, we carefully analyzed all deaths of the study participants.

### Statistical analysis and sample size calculation

Based on the data obtained by Knafelj et al. [24] in a similar population of OHCA survivors, we assumed the occurrence of the primary study end point (i.e. hospital survival with a favourable neurological outcome with CPC-1 and CPC-2), in 55% and 16% of patients treated with and without MTH, respectively. With these assumptions it was calculated before the enrolment of both control and MTH group patients that it takes at least 30 patients per group to demonstrate the significance of the difference in the occurrence of the primary study end point between patients treated with and without MTH, allowing for 90.5% power with a 2-sided alpha value of 0.05. To compensate for potential loss of study participants due to various reasons, we enrolled 5 additional patients.

The Shapiro-Wilk test was applied to verify normal distribution of the investigated continuous variables. Continuous variables were presented as mean values ± standard deviations or medians and their interquartile ranges depending on the presence or absence of normal distribution. Therefore inter-group comparisons were performed with Student’s t test for independent samples or the Mann–Whitney unpaired rank sum test when appropriate. Categorical variables were expressed as the number of patients presenting the given feature and the percentage of patients in the analysed group. Categorical variables were compared using the χ^2^ test with the Yates’ correction if required.

Univariate and multivariate logistic regression models were used to identify predictors of efficacy end points. Variables with a p-value of ≤0.1 in the univariate analysis were introduced into the multivariate logistic regression model. Subsequently, variables with no significant impact on the prevalence of efficacy end points (p ≥ 0.05) were one by one removed from the multivariate model according to their decreasing p-values. Relations between investigated variables and the likelihood of efficacy end points were estimated with the use of odds ratios (OR) with 95% confidence intervals (95% CI). Optimal cut-off points were determined using the receiver operator characteristic (ROC) analysis. Additionally, for end points for which the difference in their occurrence between patients treated with and without MTH reached statistical significance, we calculated the NNT and its 95% CI.

Two-sided differences were considered significant at p < 0.05. The statistical analysis and sample size calculation were carried out using the Statistica 10.0 package (StatSoft, Tulsa, OK, USA), while MedCalc 12.0 (MedCalc Software, Mariakerke, Belgium) statistical software was used for the ROC analysis.

## Results

### MTH procedure and patient characteristics

The MTH group comprised of 32 patients who accounted for 0.8% of the total of 4006 subjects hospitalized in our department in 2010 and 2011 due to ACS. MTH was successfully applied in all patients. The median time needed to achieve the state of MTH counted from the initiation of cooling with the infusion of cold saline and the usage of ice packs was 400 minutes, with the interquartile difference ranging between 295.0 and 666.5 minutes. None of the study participants was being cooled before hospital admission. MTH procedure was continued for a median of 24.0 (24.0-24.5) hours. Atracurium was administered in 9 (28.1%) patients due to shivers resistant to the first-line therapy.

Both groups of patients were comparable in terms of their demographic and clinical characteristics (Table 1). Although patients receiving MTH were slightly older and less frequently had a history of myocardial infarction, while those treated conservatively presented with higher scores in Glasgow Coma Scale and required shorter resuscitation to restore spontaneous circulation, all differences, except for the lower proportion of patients with the final diagnosis of ACS in the MTH group, were statistically nonsignificant. Slightly fewer patients in the MTH group underwent PCI than in the control group. Although patients in the latter group presented with more complex angiographic and procedural characteristics (more patients treated with PCI of left main coronary artery and left anterior descending artery or multi-vessel PCI, higher prevalence of type C lesions according to the American College of Cardiology/ American Heart Association classification treated with PCI, worse baseline flow in the culprit artery, lower median diameter of implanted stents), all differences between both groups were statistically nonsignificant (Table 2). Abciximab was administered during PCI in similar proportions of patients in both groups. The rate of IABP use, although higher among the controls, showed no statistically significant difference as compared with patients receiving MTH.

**Table 1:** Demographic and clinical characteristics of patients treated with and without mild therapeutic hypothermia

**Variable**	**Patients undergoing MTH procedure**	**Control group**	**p**
	**(n = 32)**	**(n = 33)**	
Demographic characteristics			
Age [years]	65.6 ± 19.0	62.7 ± 9.8	ns
Gender: men/women	25 (78.1%)/7 (21.9%)	26 (78.8%)/7 (21.2%)	ns
Medical history			
Concomitant diabetes	13 (40.6%)	13 (39.4%)	ns
Prior stroke	4 (12.5%)	3 (9.1%)	ns
Prior myocardial infarction	8 (25.0%)	13 (39.4%)	ns
Details regarding OHCA			
First-recorded cardiac rhythm			ns
Ventricular fibrillation or pulseless ventricular tachycardia	26 (81.3%)	26 (78.8%)	
Asystole	5 (15.6%)	6 (18.2%)	
Pulseless electrical activity	1 (3.1%)	1 (3.0%)	
Initiation of cardiopulmonary resuscitation by bystanders	12 (37.5%)	10 (30.3%)	ns
Time between OHCA and ROSC [minutes]	30.0 (25.0-56.0)	23.0 (18.0-37.0)	ns
Duration of cardiopulmonary resuscitation [minutes]	25.0 (15.0-51.0)	20.0 (11.0-45.0)	ns
Patient status on admission and in-hospital management			
Glasgow Coma Score on hospital admission			ns
3-4 points	22 (68.8%)	18 (54.6%)	
5-6 points	7 (21.9%)	11 (33.3%)	
7-8 points	3 (9.3%)	4 (12.1%)	
Arterial pH on hospital admission	7.26 (7.20-7.33)	7.30 (7.16-7.36)	ns
Shock on hospital admission	15 (46.9%)	14 (42.4%)	ns
LVEF on hospital admission [%]	35.0 (30.0-45.0)	30.0 (25.0-40.0)	ns
Underlying cause of OHCA			p < 0.04
STEMI	24 (75.0%)	22 (66.7%)	
NST-ACS	2 (6.2%)	9 (27.3%)	
Other	6 (18.8%)	2 (6.0%)	
Presence of coronary artery disease			ns
single-vessel	7 (21.9%)	11 (33.3%)	
multi-vessel	17 (53.1%)	20 (60.6%)	
lack of significant coronary lesions	8 (25.0%)	2 (6.1%)	
Treatment with PCI	24 (75.0%)	27 (81.8%)	ns
Use of intra-aortic balloon counterpulsation	4 (12.5%)	9 (27.3%)	ns

*LVEF* Left ventricular ejection fraction; *MTH* Mild therapeutic hypothermia; *NST*-*ACS* Non-ST-segment elevation acute coronary syndrome; *OHCA* Out-of-hospital cardiac arrest; *PCI* Percutaneous coronary intervention; *ROSC* Successful return of spontaneous circulation; *STEMI* ST-segment elevation myocardial infarction.

**Table 2:** Angiographic and procedural characteristics of the study participants undergoing percutaneous coronary intervention in relation to the treatment with mild therapeutic hypothermia

**Variable**	**Patients undergoing MTH procedure**	**Control group**	**p**
	**(n = 24)**	**(n = 27)**	
Coronary artery treated with PCI			ns
LM	0	2	
LAD	16	19	
Cx	8	8	
AL	0	1	
RCA	8	12	
Number of coronary arteries treated with PCI			ns
single-vessel PCI	17	18	
dual-vessel PCI	6	9	
triple-vessel PCI	1	2	
Number of lesions treated with PCI			ns
single-lesion PCI	13	14	
dual-lesion PCI	9	12	
triple-lesion PCI	2	1	
Type of lesion treated with PCI according to the ACC/AHA classification			ns
B1	11	7	
B2	11	9	
C	15	25	
Baseline flow in the culprit artery			ns
TIMI 0 or 1	15	20	
TIMI 2	1	1	
TIMI 3	8	7	
Final flow in the culprit artery			ns
TIMI 0	0	1	
TIMI 3	24	26	
Number of stents implanted for patient			ns
0	0	2 ^#^	
1	10	12	
2	8	8	
3 or more	5	5	
Type of implanted stent			ns
bare metal stent	42	48	
drug-eluting stent	1	0	
Total length of implanted stents per patient [mm]	33 (15–44)	33 (16–50)	ns
Diameter of implanted stent [mm]	3.3 (2.8-3.5)	3.0 (2.8-3.4)	ns
Therapy with abciximab	5 (20.8%)	5 (18.5%)	ns
Application of thrombectomy	6/24	3/27	ns
Door-to-balloon time * [minutes]	40.5 (31.5-68.5)	70.0 (32.0-81.0)	ns
Direct effect of PCI in the culprit lesion			ns
optimal	23	25	
suboptimal	1	1	
ineffective	0	1	

^#^ One patient underwent exclusively plain old balloon angioblasty while another one had an unsuccessful attempt of PCI. * In case of multi-vessel PCI door-to-balloon time refers to the culprit lesion. *ACC* American College of Cardiology; *AL* Intermediate artery; *AHA* American Heart Association; *Cx* Circumflex artery; *LAD* Left anterior descending artery; *LM* Left main coronary artery; *MTH* Mild therapeutic hypothermia; *PCI* Percutaneous coronary intervention; *RCA* Right coronary artery.

In comparison with historical control groups, we found a significant prolongation of hospitalization in patients treated with MTH (18.5 [12.5-54.0] vs. 8.0 [3.0-14.5] days; p < 0.00004). However, after exclusion of cases of in-hospital death, the difference no longer remained significant (19.0 [12.5-59.5] vs. 12.0 [10.0-22.0] days; p = ns). Therefore it seems that this difference was driven by both early deaths among patients from the control group and application of MTH procedure.

### Impact of MTH on favourable neurological outcome

**Figure 1:**
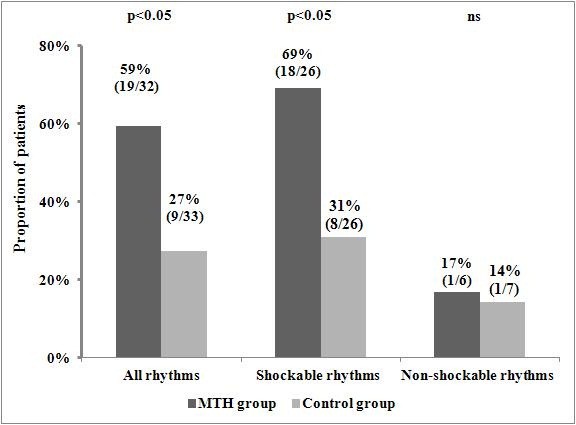
**Comparison of hospital survival with a favourable neurological outcome in patients treated with and without mild therapeutic hypothermia.** The results are reported as the percentage and the number of patients with a favourable neurological outcome of patients in the analysed group. MTH - mild therapeutic hypothermia.

**Figure 2:**
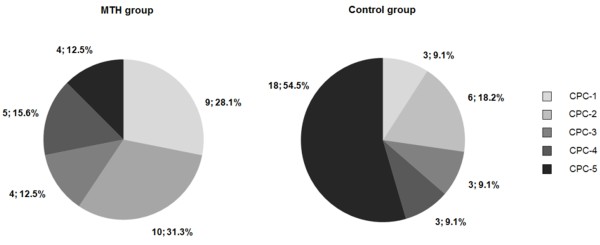
**Distribution of categories according to the Pittsburgh Cerebral Performance Category Scale in the patients undergoing mild therapeutic hypothermia and in the control group.** CPC- Cerebral Performance Category; MTH - mild therapeutic hypothermia.

A total of 28 study participants were discharged from hospital with a favourable neurological outcome. Therapy with MTH was associated with a significantly higher number of patients presenting a favourable neurological outcome both in the overall study population as well as in the sub-group of patients with shockable rhythms, but not in those with non-shockable rhythms (Figure 1). However, we did not find any difference in the proportions of patients discharged in CPC category 1 or 2 and all patients who survived until hospital discharge between subjects treated with MTH and controls in the overall study population (19/28 [67.9%] vs. 9/15 [60.0%]; p = ns) nor in the sub-groups with shockable (18/24 [75.0%] vs. 8/12 [66.7%]; p = ns) and non-shockable rhythms (1/4 [25.0%] vs. 1/3 [33.3%; p = ns]). Detailed distribution of the cerebral performance categories according to the CPC classification in patients undergoing MTH and controls is displayed in Figure 2. Values of NNT for the achievement of a favourable neurological outcome for the overall study population and for the sub-group of patients with ventricular fibrillation or pulseless ventricular tachycardia were 3.11 (95% CI 1.82-10.73) and 2.90 (95% CI 1.65-12.25), respectively. A direct comparison of characteristics of patients with and without in-hospital favourable neurological outcome including all variables listed in Table 1 revealed significant differences concerning patients’ age (54.4 ± 11.5 vs. 66.2 ± 9.6 years; p < 0.00004); the duration of cardiopulmonary resuscitation (18.5 [9.0-30.0] vs. 25.0 [20.0-57.0] minutes; p < 0.04), proportion of patients with Glasgow Coma Score > 3 points on hospital admission (21/28 [75.0%] vs. 17/37 [45.9%]; p < 0.02) and the proportion of patients treated with MTH (19 [67.9%] vs. 13 [35.1]; p < 0.01). Additionally, logistic regression analysis (both univariate and multivariate) indicated a significant prognostic value of MTH therapy in terms of predicting a favourable neurological outcome (Table 3). Logistic regression-based calculations also identified younger age as a powerful predictor of in-hospital survival with a favourable neurological outcome with the highest diagnostic accuracy at the cut-off value of ≤56.0 years of age as calculated in the ROC curve analysis (sensitivity 57.1%, specificity 86.5%, positive predictive value 76.2%, negative predictive value 72.7%). The area under the ROC curve for patients’ age was 0.69 (95% CI 0.63- 0.76).

**Table 3:** Predictors of hospital survival with a favourable neurological outcome in univariate and multivariate analysis

**Univariate analysis**			
	**OR**	**95% CI**	**p**
OHCA with shockable rhythm	5.50	1.07-28.16	p < 0.05
Treatment with MTH	3.90	1.35-11.27	p < 0.02
Glasgow Coma Score on hospital admission [patients with >3 points vs. patients with 3 points]	3.53	1.18-10.53	p < 0.02
Prior myocardial infarction	0.38	0.12-1.20	p = 0.097
Age [for a 10 year increase]	0.34	0.18-0.63	p < 0.001
Multivariate analysis			
	OR	95% CI	p
Treatment with MTH	4.13	1.20-14.19	p < 0.03
Age [for a 10 year increase]	0.33	0.17-0.64	p < 0.002

Results are presented according to decreasing values of odds ratios.

*CI* Confidence interval; *MTH* Mild therapeutic hypothermia; *OHCA* Out-of-hospital cardiac arrest; *OR* Odds ratio.

### Impact of MTH on all-cause in-hospital mortality

**Figure 3:**
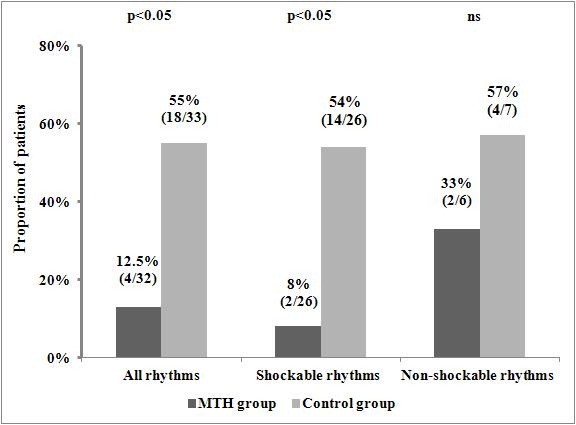
**Comparison of all-cause in-hospital mortality in patients treated with and without mild therapeutic hypothermia.** The results are reported as the percentage and the number of patients who died during the index hospitalization in the analysed group. MTH - mild therapeutic hypothermia.

A total of 22 study participants died during the index hospitalization. As shown in Figure 3, patients undergoing MTH when compared with the control group experienced remarkably lower all-cause in-hospital mortality (NNT = 2.38; 95% CI 1.60-4.67). The difference was even more pronounced in those with shockable rhythms (NNT = 2.21; 95% CI 1.47-4.53). A direct comparison of characteristics of cases of in-hospital deaths and survivals including all variables listed in Table 1 revealed significant differences regarding patients’ age (66.2 ± 8.6 vs. 58.4 ± 12.6 years; p < 0.02); proportion of patients with Glasgow Coma Score > 3 points on hospital admission (8/22 [36.4%] vs. 30/43 [69.8%] points; p < 0.01) and the proportion of OHCA patients treated with MTH (4 [18.2%] vs. 28 [65.1%]; p = 0.0009). Furthermore, both univariate and multivariate analyses identified the lack of MTH therapy as a significant predictor of all-cause in-hospital mortality (Table 4). In addition to the previous, elderly age was also independently associated with the risk of in-hospital death, with the area under the curve of 0.67 as revealed by a ROC curve analysis to assess the diagnostic accuracy for the prediction of all-cause in-hospital mortality (95% confidence interval 0.54-0.78; p < 0.01). The corresponding optimal cut-off value was >56.0 years of age (sensitivity 90.9%, specificity 44.2%, positive predictive value 45.5%, negative predictive value 90.5%).

**Table 4:** Predictors of all-cause in-hospital mortality in univariate and multivariate analysis

**Univariate analysis**			
	**OR**	**95% CI**	**p**
Age [for a 10 year increase]	1.88	1.11-3.18	p < 0.02
LVEF on admission [for a 10% increase]	0.56	0.30-1.06	p = 0.074
Glasgow Coma Score on hospital admission [patients with >3 points vs. patients with 3 points]	0.25	0.08-0.73	p < 0.02
Treatment with MTH	0.12	0.03-0.43	p < 0.002
Multivariate analysis			
	OR	95% CI	p
Age [for a 10 year increase]	2.09	1.10-3.96	p < 0.03
Treatment with MTH	0.11	0.03-0.43	p < 0.003

*CI* Confidence interval; *LVEF* Left ventricular ejection fraction; *MTH* Mild therapeutic hypothermia; *OR* Odds ratio.

Results are presented according to decreasing values of odds ratios.

### Safety of MTH therapy

Neither definite nor probable stent thrombosis occurred in MTH-treated patients. Similarly, none of the patients from the control group developed definite stent thrombosis, while 2 patients from this group were classified as having probable stent thrombosis. These patients died suddenly on the 2nd and 4th day of hospitalization, respectively, despite their relatively stable condition and there was no clear explanation for their deaths. Unfortunately, in both cases autopsy was not performed. Despite high absolute rates of pneumonia, we found no difference in respect to the primary safety end point between patients treated with and without MTH (Table 5). We also noted similar proportions of subjects with positive blood cultures in both groups. Similarly, the rates of bleeding complications as well as rhythm and conduction disorders did not differ between patients undergoing MTH and controls. However, the incidence of bleedings of any severity and red blood cell transfusions was numerically higher in patients treated with MTH. Cardioverter-defibrillators were implanted during the index hospitalization in 7 patients (21.9%) in the MTH group and in only 1 patient (3.0%) in the control group (p = 0.053). None of the study participants underwent pacemaker implantation during the index hospitalization.

**Table 5:** Comparison of the occurrence of safety end points in patients treated with and without mild therapeutic hypothermia

	**Patients undergoing MTH procedure**	**Control group**	**p**
	**(n = 32)**	**(n = 33)**	
Definite stent thrombosis*	0 (0%)	0 (0%)	ns
Probable stent thrombosis*	0 (0%)	2 (7.4%)	ns
Pneumonia	19 (59.4%)	19 (57.6%)	ns
Positive blood culture	12 (37.5%)	11 (33.3%)	ns
Bleeding of any severity	8 (25.0%)	5 (15.2%)	ns
Red blood cell transfusion	4 (12.5%)	2 (6.1%)	ns
Rhythm and conductions disorders:			ns
Atrial fibrillation	6 (18.8%)	7 (21.2%)	
Ventricular tachycardia	3 (9.4%)	1 (3.0%)	ns
Clinically relevant bradycardia	3 (9.4%)	1 (3.0%)	ns

* The percentage refers to the number of patients treated with percutaneous coronary intervention in the group. *MTH* Mild therapeutic hypothermia.

## Discussion

According to our best knowledge this study is the first European report proving that implementation of an MTH program for the treatment of comatose OHCA survivors based on a previously established ACS network improves clinical outcomes. Additionally, our data indicate that a standardized MTH protocol may be successfully developed and used by cardiologists trained in acute cardiac care. Our study together with the first overall experience from the Minneapolis Heart Institute [19] strongly supports the concept of implementation of MTH programs in centres which provide interventional treatment for ACS patients. The clinical effectiveness of MTH, namely the enormous improvement in neurological status and striking reduction in all-cause in-hospital mortality among patients treated with this method when compared with a historical control group, was even greater in our study than in randomized clinical trials [6-9]. Of major importance, according to our data, treatment with MTH was not accompanied by any excess of complications including stent thrombosis, bleedings and infections as well as rhythm and conductions disorders.

Organization of regional systems of care for comatose OHCA survivors is robustly encouraged by international experts [13,25,26]. In support of the recommendation on the development of regional systems of care for OHCA patients, an analysis of a Japanese registry including 10,383 patients demonstrated that the rate of neurologically favourable 1-month survival after OHCA of presumed cardiac aetiology in patients transported to critical cardiac care hospital was higher than in those transported to hospitals without specialized cardiac facilities (6.7 vs. 2.8%; p < 0.001) [27]. In line with this observation, a recent prospective cohort study of the Resuscitation Outcomes Consortium (ROC) suggested better survival to discharge if closer, but inadequately equipped hospitals were bypassed in favour of further hospitals with more advanced therapeutic capabilities (16.5 vs. 12.1%) [28]. Furthermore, the ROC investigators noted higher survival rates to discharge in hospitals performing cardiac catheterization (34 vs. 27%; p = 0.001) and in hospitals with at least 40 OHCA patients per year (37 vs. 30%; p = 0.01) [29]. Finally, Mooney et al., who for the first time superimposed an MTH program for the treatment of comatose patients, demonstrated an overall survival to hospital discharge rate of 56% among 140 study participants, with 92% of survivors discharged in the CPC category of 1 or 2 [20]. Three quarters of the patients (n = 107) in this study were transferred to the MTH-capable hospital from referring network hospitals. Whether transferred or not to the MTH-capable centre, the patients showed similar survival rates. Although both, our study and the original one, refer to the same clinical scenario, they differ in several aspects. Not only is our series much smaller than the report from Minneapolis Heart Institute, but it also indicates a lower rate of bystander cardiopulmonary resuscitation (38 vs. 66%), longer time from cardiac arrest to the return of spontaneous circulation (30 vs. 22 minutes), and higher overall survival rate at hospital discharge (87 vs. 56%). Surprisingly, we noted a higher proportion of patients surviving with significant neurologic impairment in the MTH group (32.1%) than in most reports including the study conducted by Mooney et al. [6-9,20]. However, this potentially alarming finding should be interpreted with caution due to the limited sample size of our study and the true occurrence of poor neurologic outcome among OHCA survivors treated with MTH should be carefully evaluated in large multicentre registries.

Our ACS network was developed in 2002 in order to provide optimal care for ACS patients in the Kuyavia and Pomerania Region, particularly aiming to identify high-risk ACS subjects to enable their immediate transportation to experienced PCI centres. The implementation of a standarized MTH program in 2010, complements the rest of modalities employed so far in the care of OHCA survivors, such as mechanical ventilation, circulatory support with IABP and invasive and non-invasive techniques of haemodynamic status monitoring. Based on this evidence supporting the beneficial impact of MTH in post-OHCA patients, we find it reasonable to further encourage health care providers from our region in more active participation in our system of care for OHCA survivors. Patients treated with MTH in our study accounted for merely 0.8% of all ACS patients hospitalized in our department in 2010 and 2011 and it is likely that even currently a large number of OHCA patients are still being admitted to general intensive care units without MTH facilities.

In our opinion, the following factors, absent or under-utilized in randomized clinical trials, including administration of cold intravenous infusions preceding endovascular cooling, routine performance of coronary angiography combined, if needed, with PCI in all OHCA survivors with the initial ACS diagnosis, multidisciplinary specialist care and use of continuous monitoring of invasive blood pressure and central venous pressure might potentially contribute to the extent of clinical benefits observed in our MTH group. Although the MTH and control groups in our study were similar in terms of their characteristics, it is likely that some of these factors may exert a synergistic effect to that attributed to the treatment with MTH.

In contrast to the latest recommendation of the European Resuscitation Council Guidelines to use MTH in comatose OHCA survivors with initially non-shockable rhythms [29], we failed to prove any benefits of MTH in this particular population. Although our study was underpowered to assess clinical outcomes in this sub-group, our results correspond with those obtained in numerous [30-32], but not all [8,33,34], previous observational and small randomized studies. As a consequence of inconclusive evidence, the guidelines acknowledge a lower level of evidence for the use of MTH after cardiac arrest from non-shockable rhythms than from shockable ones [12]. OHCA patients with non-shockable rhythms have much poorer outcomes than their counterparts with shockable rhythms, particularly due to more comorbidities and frequently longer periods from the OHCA occurrence to the initiation of resuscitation [4]. Therefore it is likely that MTH may be insufficient to reverse the unfavourable prognosis in a substantial proportion of OHCA victims with non-shockable rhythms. To clarify the efficacy of MTH in patients with non-shockable rhythms, an adequately designed randomized trial in this population is warranted.

The presence of coronary artery disease in up to 71% of OHCA victims together with acute coronary artery occlusion in nearly a half of them provides a solid rationale for the performance of coronary angiography in these patients [35]. These numbers reported by Spaulding et al. correspond with our data. Of interest, the absence of ST-segment elevation on the surface 12-lead ECG after cardiopulmonary resuscitation does not guarantee coronary vessels patency. It is even estimated that approximately 20-30% of patients after OHCA present with coronary artery occlusion or an unstable coronary lesion, despite the lack of ST-segment elevation [36,37]. An analysis of Arizona SHARE database indicates an underlying noncardiac aetiology in only 20% of adult nontraumatic OHCA cases [38]. Numerous recent reports demonstrate substantial advantages of coronary revascularisation in patients after OHCA [24,36,39,40]. For example, in the PROCAT (*Parisian Region Out of Hospital Cardiac Arrest*) registry successful PCI was demonstrated to be an independent predictor of survival, regardless of the postresuscitation ECG pattern (odds ratio 2.06; 95% confidence interval 1.16-3.66) [36]. Therefore, we believe that combining early coronary angiography and PCI with timely induction of MTH should be considered an optimal strategy in this setting. However, we need to facilitate pre-selection of patients, particularly those without ST-segment elevation, benefiting from PCI after OHCA to avoid unnecessary coronary angiographies. Among 435 patients with a cardiac cause of OHCA included in the above mentioned PROCAT registry, ST-segment elevation was present in 134 subjects while the rest of participants presented with other ECG patterns. Successful PCI was completed in 74% and 26% of patients in the former and in the latter group, respectively.

Of major importance, a recent case series including 11 patients treated with MTH and coronary stenting (13 bare-metal stents and 3 drug-eluting stents) indicated an unexpectedly high rate of in-hospital stent thrombosis (45.5%), despite the periprocedural administration of abciximab in 6 patients including 5 of 6 subjects with stent thrombosis [20]. In 4 patients, stent thrombosis was diagnosed during coronary angiography, and in the last one, the diagnosis was made at autopsy. When compared with non-OHCA patients undergoing coronary stenting due to ACS in the routine clinical practice, the rate of stent thrombosis reported by Penela et al. remains extremely high (1–3 vs. 45,5%) even considering the phenomena of MTH-related increased ADP-induced platelet aggregation and impaired bioavailability and antiplatelet effect of clopidogrel in OHCA patients [20,41-43]. In contrast to this observation, we found in our data no cases of stent thrombosis in MTH-treated patients. It might be partially attributed to doubling the clopidogrel maintenance dose and ongoing therapy with unfractionated heparin in our study group but the possible excess of stent thrombosis in the MTH settings warrants further investigation.

As stated in the guidelines of European Society of Cardiology for the management of acute myocardial infarction in patients presenting with ST-segment elevation the optimal sequence of cooling and PCI in OHCA survivors is unclear [13]. Preclinical studies and small clinical trials suggested that MTH if obtained before reperfusion may reduce infarct size [44,45]. However, the hypothesis regarding clinical benefits of MTH in non-OHCA patients with myocardial infarction warrants further investigation. This approach requires dedicated local techniques such as transcoronary induction of MTH using a catheter or wire lumen to limit the cooling area to myocardium in conscious patients undergoing primary PCI. Although the induction of MTH was initiated in our study in the cathlab before coronary angiography, it is unlikely that the clinical benefits of MTH in our study were related to the protective effect of cooling on myocardium since the median time needed to achieve the state of MTH was as long as 400 minutes.

Available evidence from animal models and small case series of human patients suggest an advantageous effect of MTH in the setting of post-myocardial infarction cardiogenic shock [46,47]. German researchers, who compared 20 consecutive patients admitted in cardiogenic shock after successful resuscitation from OHCA and treated with MTH with 20 matched subjects from a historic normothermic control group, observed in the former a significant decrease in heart rate and marked increase in ejection fraction, mean arterial pressure and systemic vascular resistance, with no change in cardiac index [47]. Accordingly, patients from the MTH group required lower cumulative doses of vasopressors and inotropes. Other potentially beneficial physiologic effects of MTH in cardiogenic shock include lower oxygen consumption along with protection from myocardial damage and reduction of end-organ injury from prolonged hypoperfusion [46]. In our study, cardiogenic shock on admission was present in 46.9 and 42.4% of patient treated with and without MTH, respectively. Surprisingly, the presence of cardiogenic shock on admission did not carry an adverse prognosis in our study but it should be emphasized that all study participants underwent invasive management and patients with ongoing hemodynamic instability (systolic arterial pressure below 65 mm Hg), despite therapy with vasopressors and/or application of IABP, were not eligible for MTH treatment unless they achieved hemodynamic stabilization. On the other hand, we cannot exclude that this observation may be attributed to the favourable impact of MTH. However, the efficacy and safety of MTH in patients with cardiogenic shock needs to be verified in adequately designed randomized trials.

The HACA (the Hypothermia after Cardiac Arrest Study) investigators found a numerically higher, but statistically insignificant, prevalence of pneumonia in the MTH group than in the normothermia group [6]. It raised concerns regarding the safety of the method. A meta-analysis conducted by Arrich et al. did not confirm the detrimental effect of MTH on the occurrence of pneumonia and other adverse events [16]. These findings are in line with our results. However, all trials included in this meta-analysis, similarly to our study, were underpowered to show modest differences in the prevalence of safety end points. On the other hand, pneumonia occurred in almost 60% of our study participants which is comparable with other studies [48,49]. Therefore, pneumonia, usually caused by aspiration or mechanical ventilation, remains the leading complication with paramount clinical importance in comatose OHCA survivors.

### Study limitations

Several limitations of our study should be acknowledged. First, despite the fact that the MTH and control groups were relatively well-matched, the non-randomized and retrospective design of our study may result in overestimation of benefits related to MTH therapy. Second, the limited sample sizes of our study preclude comprehensive sub-group analyses and substantially reduce the possibility to perform complex multivariate analyses. Third, the stable care assumption, inherent to the choice of historical controls, should be mentioned as an another important limitation of our study. Fourth, our observation is restricted to the in-hospital period. Although neurologic status at hospital discharge and all-cause in-hospital mortality constitute common endpoints in MTH studies, prolongation of the follow-up would definitely increase the value of our findings. Fifth, differences in the underlying cause of OHCA between patients treated with and without MTH as well as in door-to-balloon times, namely preponderance of patients with ST-segment elevation myocardial infarction and non-coronary OHCA aetiology in the MTH group and non-ST-segment elevation ACS subjects in the control group, together with shorter door-to-balloon time in the former, might have influenced the results. Sixth, the relatively long time needed to achieve the state of MTH in our study, despite the implementation of the endovascular cooling system, might possibly attenuate clinical advantages derived from MTH therapy. It was caused by the initiation of endovascular cooling in Cardiac Intensive Care Sub-unit after the completion of coronary interventional management. Additionally, none of the study participants was being cooled before hospital admission and no ice packs were attached to the patients’ chest during coronary angiography and PCI to avoid adverse effects on the quality of images. Seventh, there was a statistically non-significant discrepancy concerning the use of IABP support in patients with cardiogenic shock between the MTH and control groups. It was driven by the recent critical appraisal of IABP treatment in this setting and a gradual decrease in IABP utilization in routine practice due to the lack of proven clinical benefits associated with this therapy [50].

## Conclusions

Our study indicates that a regional system of care for OHCA survivors may be successfully implemented based on an ACS network, leading to an improvement in neurological status and to a reduction of in-hospital mortality in patients treated with MTH, without any excess of complications. However, our findings should be verified in large, prospective trials.

## Competing interests

Dr. Krzysztof Pstrągowski received a speaker fee from Polimed Sp. z o.o. company, the distributor of the CoolGard 3000 system in Poland. The rest of the authors declare that they have no competing interests.

## Authors’ contributions

MK: design, data collection, analysis and interpretation, drafting and revision of the manuscript. KP: conception, design, data collection and interpretation, drafting and revision of the manuscript. JMK: data collection and interpretation, revision of the manuscript. TF: drafting and revision of the manuscript. MK: data analysis and interpretation, revision of the manuscript. BK: data collection and interpretation, revision of the manuscript. PP: conception, data interpretation, revision of the manuscript. EPN: data analysis and interpretation, drafting and revision of the manuscript. GG: drafting and revision of the manuscript. JK: conception, design, data interpretation, drafting and revision of the manuscript. All authors read and approved the final manuscript.
